# Co-Occurrence of β-Lactam and Aminoglycoside Resistance Determinants among Clinical and Environmental Isolates of *Klebsiella pneumoniae* and *Escherichia coli*: A Genomic Approach

**DOI:** 10.3390/ph15081011

**Published:** 2022-08-17

**Authors:** Hisham N. Altayb, Hana S. Elbadawi, Faisal A. Alzahrani, Othman Baothman, Imran Kazmi, Muhammad Shahid Nadeem, Salman Hosawi, Kamel Chaieb

**Affiliations:** 1 Department of Biochemistry, Faculty of Science, King Abdulaziz University, Jeddah 21589, Saudi Arabia; 2 Centre for Artificial Intelligence in Precision Medicine, King Abdulaziz University, Jeddah 21589, Saudi Arabia; 3 Microbiology and Parasitology Department, Soba University Hospital, University of Khartoum, Khartoum 11115, Sudan; 4 Embryonic Stem Cells Unit, King Fahd Medical Research Center, King Abdulaziz University, Jeddah 21589, Saudi Arabia; 5 Laboratory of Analysis, Treatment and Valorization of Pollutants of the Environmental and Products, Faculty of Pharmacy, University of Monastir, Monastir 5000, Tunisia

**Keywords:** mobile genetic elements, AMR, ESBLs, whole genome sequencing, MDR

## Abstract

The presence of antimicrobial-resistance genes (ARGs) in mobile genetic elements (MGEs) facilitates the rapid development and dissemination of multidrug-resistant bacteria, which represents a serious problem for human health. This is a One Health study which aims to investigate the co-occurrence of antimicrobial resistance determinants among clinical and environmental isolates of *K. pneumoniae* and *E. coli*. Various bioinformatics tools were used to elucidate the bacterial strains’ ID, resistome, virulome, MGEs, and phylogeny for 42 isolates obtained from hospitalized patients (*n* = 20) and environmental sites (including fresh vegetables, fruits, and drinking water) (*n* = 22). The multilocus sequence typing (MLST) showed that *K. pneumoniae* belonged to ten sequence types (STs) while the *E. coli* belonged to seventeen STs. Multidrug-resistant isolates harbored β-lactam, aminoglycoside resistance determinants, and MGE were detected circulating in the environment (drinking water, fresh vegetables, and fruits) and in patients hospitalized with postoperative infections, neonatal sepsis, and urinary tract infection. Four *K. pneumoniae* environmental isolates (7E, 16EE, 1KE, and 19KE) were multidrug-resistant and were positive for different beta-lactam and aminoglycoside resistance determinants. *bla_CTX-M-15_* in brackets of ISEc 9 and Tn 3 transposases was detected in isolates circulating in the pediatrics unit of Soba hospital and the environment. This study documented the presence of bacterial isolates harboring a similar pattern of antimicrobial resistance determinants circulating in hospitals and environments. A rapid response is needed from stakeholders to initiate a program for infection prevention and control measures to detect such clones disseminated in the communities and hospitals.

## 1. Introduction

Antimicrobial resistance in Enterobacterales, especially *K. pneumoniae* and *E. coli*, is a critical threat to public health [1,2]. *K. pneumoniae* and *E. coli* contribute to the rapid evolution of antibiotic-resistance genes due to genomic plasticity [3]. They have the ability to acquire, accumulate, and disseminate the MGEs associated with antimicrobial resistance such as plasmids and transposons [4,5]. Studying MDR mechanisms and the sequencing of ARGs in these organisms is critical to understanding both the molecular mechanisms of resistance and the basis of their dissemination [6,7].

There is a growing concern regarding controlling the global development and spreading of antibiotic-resistant pathogens, especially for MDR bacteria that produce Extended Spectrum β-Lactamases (ESBLs) and carbapenemases [8]. β-lactam antibiotics represent the most common drug class of antimicrobial drugs with broad clinical implications [9]. The β-lactamases produced by the Enterobacteriaceae family, particularly *K. pneumoniae* and *E. coli*, are hydrolytic enzymes that confer bacterial resistance to β-lactam antibiotics such as penicillin, cephalosporin, and carbapenem families that are common antimicrobial drugs used all around the world [2]. Nosocomial MDR *K. pneumoniae* and *E. coli* have been considered the most frequent agents of infectious diseases and significant threats to patients in hospital settings in Sudan [10,11,12]. The presence of antimicrobial-resistance genes in MGEs of the environment and clinical strains facilitate the rapid development and dissemination of MDR bacteria and thus represent a serious problem for human health [13]. Different transposable elements are associated with the spread of antimicrobial-resistance genes between bacteria, including plasmids, transposons, and integrons [14]. There are many plasmids associated with ARGs in the Enterobacterales group (e.g., IncF, IncA/C, IncH, IncP, IncL/M, or IncX, etc.). The IncF plasmid is more frequently described as carrying genes encodes for resistance to ESBLs, carbapenems, aminoglycosides, or fluoroquinolones, while IncI2, IncX4, and IncP plasmids encode for gene resistance to colistin. The IncHI1 and IncHI2 plasmids are more frequently reported in MDR isolates [15]. Drug-resistant bacteria can be transferred to humans from the surrounding contaminated environment, including soil, animals, water, vegetables, and fruits [16,17]. The contamination of drinking water and daily consumable products with antimicrobial-resistant bacteria represent a serious problem due to their ease of transmission to human settlements, hospitals, and crowded areas [18,19]. The spread of MDR bacteria in the environment and health settings has led to increased mortality and morbidity rates and is now considered one of the most serious public health threats [20]. Khartoum is one of the most crowded, polluted cities in Africa [21,22], which represents a suitable medium for the dissemination of antimicrobial-resistant bacteria. Sudan suffers from the inappropriate use of antibiotics; most of the antibiotics are frequently sold over the counter, and even without a medical prescription [23,24]. Recently, different studies conducted in Khartoum state have documented the dissemination of antibiotic-resistant bacteria in the environment such as banknotes [25] and drinking water [26,27]. Whole-genome sequencing (WGS) and plasmid analysis are essential for accurate epidemiologic tracking of hospital outbreaks and routine surveillance. Recently, WGS has played an important role in speeding up microbial diagnosis and predicting antimicrobial resistance [28]. There is no information regarding the phenotypic and genetic characteristics of the environmental MDR *Klebsiella* spp. and *E. coli* isolates and their relation to nosocomial isolates. The present study aims to characterize and to identify the MGEs, antimicrobial-resistance genes, and STs of *K. pneumoniae* and *E. coli* spreading in clinical and environmental isolates using WGS.

## 2. Results

### 2.1. Isolates

A total of 42 isolates were identified and confirmed as *K. pneumoniae* and *E. coli* by phenotypic tests and the PubMLST database. The isolates were as follows: clinical isolates (*K. pneumoniae* = 7, *E. coli* = 13) and environmental isolates (*K. pneumoniae* =13, *E. coli* = 9) (Table 1).

### 2.2. Phenotypic Antimicrobial Susceptibility Testing

Antimicrobial susceptibility testing revealed variation in the resistance patterns (Table 2); all studied isolates were susceptible to carbapenems, while some isolates were resistant to beta-lactam, aminoglycoside, and ciprofloxacin (Table 2). Sequencing results confirmed the presence of different types of ARGs. All (100%) clinical and environmental *K. pneumoniae* isolates (*n* = 20) harbored beta-lactam, aminoglycoside, and fluoroquinolones resistance genes while 13 (59%) of *E. coli* clinical and environmental isolates (*n* = 22) harbored beta-lactam, aminoglycoside, and fluoroquinolones, and about 72% harbored other resistance genes (Table 2).

### 2.3. Genomic Sequence Features and Strains Typing

All assembled sequences had greater than 88× coverage, the *E. coli* had an average GC content of 50.65%, N50 of 106319, 395 contigs, and coding sequences (CDS) of 4914. *K. pneumoniae* had an average GC content of 57.16%, N50 of 158435, 249 contigs, and 5414.3 CDS (Supplementary File, Table S1).

The MLST showed that *K. pneumoniae* isolates belonged to ten STs, while the *E. coli* isolates belonged to seventeen STs. ST45 was detected in eight *K. pneumoniae* isolates (six were clinical and two environmental isolates) and ST405 was detected in five clinical *E. coli* isolates. The most common strain of *E. coli* in urinary tract infection (UTI) patients was ST405, while *K. pneumoniae* ST45 was the most common in patients with septicemia. The list of all different STs was presented in Table 1. One *K. pneumoniae* isolate (23KE) showed one novel allele (phoE_4) in the *phoE* gene and was assigned a novel sequence type with ID:1504, while isolates 16KE and 17KE were identified with novel alleles and assigned a novel sequence type with IDs, 22233 and 22234. Isolate 14EE was submitted to the Pasteur MLST database and assigned with the ID: 1508.

### 2.4. Detection of Antibiotic-Resistance Genes (ARGs)

Analysis of resistome revealed that 40% (17/42) of the isolates harbored one or more of the aminoglycoside-resistant genes (*aph(6)-Id* and *aph(3″)-Ib*, *aac(6′)-Ib-cr, aadA5*, and *rmtB*). These genes were detected in ten *E. coli* isolates (one environmental and nine clinical) and seven *K. pneumoniae* isolates (one environmental and six clinical).

The β-lactam-resistant determinants were detected in 79% (33/42) of the isolates including 13 *E. coli* (2 environmental and 11 clinical) and 20 *K. pneumoniae* (13 environmental and 7 clinical).

*K. pneumoniae* isolates possessed *ompK37* 19 (95%)*, ompK36* 17(85%), and *bla*_SHV_ group 18 (90%), and other β-lactamases including *bla*_CTX-M-15_ (*n* = 7), *bla*_OXA-1_ (*n* = 6), and *bla*_TEM-1D_ (*n* = 2). Aminoglycoside-resistant genes (*aac(6′)-Ib-cr* and *aac(3)-IIa*) were present in seven *K. pneumoniae* isolates. The *OqxB*, *OqxA*, and *acrR* genes contributing to fluoroquinolones resistance were detected together in 18 *K. pneumoniae* isolates. Analysis of resistome associated with *E. coli* isolates revealed that *blaCTX-M-15* was the most dominant gene (*n* = 8). Other β-lactamases including *bla*_NDM-5_ (*n* = 4), *bla*_OXA-1_ (*n* = 4), *bla*_TEM-1D_ (*n* = 5), *bla*_TEM-35_ (*n* = 2), *bla*_CMY_ (*n* = 2), *bla*_DHA-1_ (*n* = 1), and *bla*_SHV-12_ (*n* = 1) were also detected. Aminoglycoside- and fluoroquinolone-resistant genes were detected in *E. coli* isolates: *aac(6*′*)-Ib-cr*, *aph(3*″*)-Ib*, *mdf(A)*, *qnrB4*, *rmtB*, and *qepA4*. Genes resistant to fosfomycin, tetracycline, macrolides, and trimethoprim were identified in both *K. pneumoniae* and *E. coli*, and they include: *fosA*, *tet(A)*, *tet(B)*, *catB3*, *dfrA12*, *dfrA14*, *dfrA17*, *floR sul1*, *sul2*, and *mph(A)*, in addition to efflux genes such as *qacE* and *sitABCD* (Table 3).

Regarding the relationship of isolate sequence types (STs) with the presence of resistant genes, six clinical *K. pneumoniae* ST45 were reported with different resistant genes (*bla*_OXA-1_, *bla*_CTX-M-15_, *bla*_SHV-1_, *ompK37*, and *ompK36*). Four environmental strains of *K. pneumoniae* (1KE, 12KE, 17KE, and 21KE) were harboring *bla*_SHV-11_. Other *bla*_SHV_ variants including *bla*_SHV-1,_ *bla*_SHV-26_, *bla*_SHV-38_, and *bla*_SHV-71_ were also reported in environmental strains (Table 3). *bla*_NDM-5_ and *bla*_CTX-M-15_ genes were most common in *E. coli* ST405 strains. Four environmental isolates (7E, 16EE, 1KE, and 19KE) were MDR and were positive for different beta-lactamase genes (Table 3). The *K. pneumoniae* strain (ST1504) was isolated from drinking water at the Khartoum locality, and it was positive for *bla*_SHV-1_, *ompK37*, *ompK36*, *acrR, OqxB*, *OqxA*, and *fosA* genes. Mutations associated with fluoroquinolone and fosfomycin resistance were investigated, and amino acid substitutions were reported in genes involved in resistance to fosfomycin (*cyaA, UhpT,* and *GlpT*) and fluoroquinolone (*parC, gyrA,* and *marR*). The *cyaA* gene mutation (S352T) was observed in eight clinical isolates of *E. coli*; *UhpT* (E350Q) was observed in two isolates of *E. coli* (clinical and environment) and seventeen of *K. pneumoniae*. The *GlpT* (E448K) was present only in 20 *E. coli* isolates (90%). The *parC* (S80I) and *gyrA* (D87N, S83L) were reported only in eight *E. coli* isolates. Mutations (Y137H, G103S) in the *marR* gene were common in *E. coli*, reported in 15 isolates (Table S2). Genes associated with antibiotic efflux, antibiotic target alteration, and protection were also investigated; nine efflux pump genes (*acrB*, *emrB*, *mdtG*, *AcrE*, *cpxA*, *evgA*, *mdtE*, *TolC*, and *mdtH*) were dominantly and exclusively reported in *E. coli*, while in *K. pneumoniae LptD*, *oqxA*, *K. pneumoniae KpnF*, *K. pneumoniae KpnH*, *K. pneumoniae KpnG*, *adeF*, and *CRP*, efflux pump genes were dominant and exclusive. Most isolates of *K. pneumoniae* harbored *ArnT* (19 isolates) and *eptB* (16 isolates) genes, which are associated with antibiotic target protection (Table S3).

### 2.5. Analysis of Mobile Genetic Elements (MGEs)

Regarding the analysis of MGEs (plasmid, transposases, and virulence factors), they were present in most clinical isolates with few in environmental strains. The most prevalent plasmid in *E. coli* isolates were IncFIA (*n* = 12), IncFIB (AP001918) (*n* = 11), Col (BS512) (*n* = 9), and IncY (*n* = 6), while the prevalent *K. pneumonia-*encoding plasmids were IncFIB(K) (*n* = 12), IncFII(K) (*n* = 9), and Col440I (*n* = 10). Table 4 presents the distributions of plasmids on the study isolates; more details about transposases and virulence genes are in the additional file, Table S4.

### 2.6. Co-Occurrence of ARGs with Transposases

**Figure 1:**
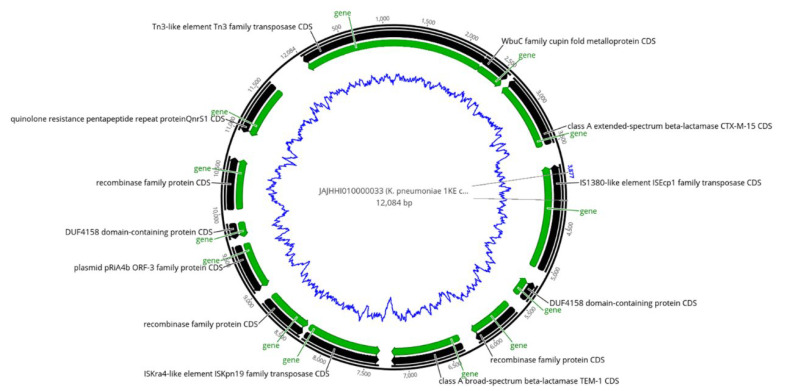
Map of different antibiotic-resistance genes, transposases, and plasmid, clustered in contig 33 of the *K. pneumoniae* (1KE) environmental strain. Showing an example of the presence of the *CTX-M-15* gene which is located between two transposases. The outer black circle indicates the contig length, black arrows indicate coding sequences (CDS), green arrows indicate genes, and the inner blue zigzag circle indicates GC content.

**Figure 2:**
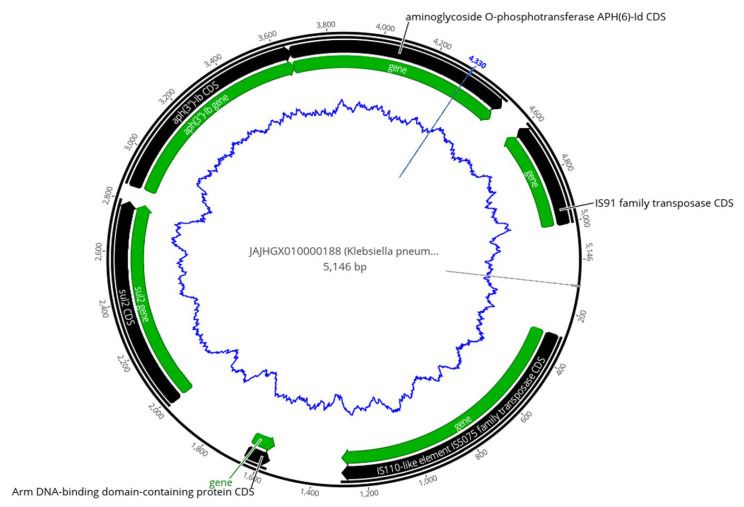
Map of antibiotic resistance and transposases cassette, identified in contig 188 of clinical *K. pneumonia* (14KP), showing the aminoglycoside-resistant genes flanked by three transposase genes. The outer black circle indicates the contig length, black arrows indicate coding sequences (CDS), green arrows indicate genes, and the inner blue zigzag circle indicates GC content.

The mobile element finder revealed the co-occurrence of ARGs, insertion sequences, transposons, and plasmids, at both environmental and clinical isolates. Some genes clustered together at the same contig (Table S5). *blaCTX-M-15* was located on contigs bracketed by *ISEc9* and *Tn3* transposases, and detected in seven isolates; one clinical *E. coli* (8EP) isolate and six *K. pneumoniae*, one environmental (1KE) (Figure 1) and five clinical isolates (5KP, 6KP, 7KP, 13KP, and 14KP) (Figures S1–S5), were detected from patients with septicemia (four of them were neonates). They were characterized by the presence of sulfonamide-resistant dihydropteroate synthase genes (*Sul2*) and aminoglycoside-resistant genes (*aph(6)-Id* and *aph(3*″*)-Ib*) bracketed by IS5075 and IS91 insertion sequences (Table S5 and Figure 2).

**Figure 3:**
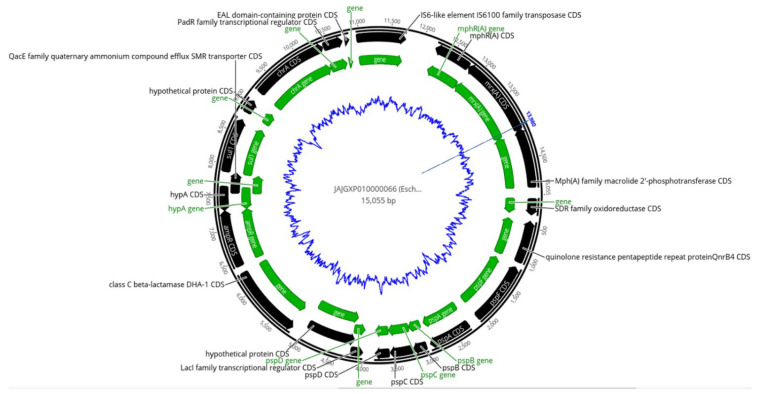
Map of ARGs and IS6 transposase cassette, identified in contig 66 of *E. coli* (1EP). The outer black circle indicates the contig length, black arrows indicate coding sequences (CDS), green arrows indicate genes, and the inner blue zigzag circle indicates GC content.

As shown in Figure 3, IS6100 clustered with ARGs and was observed similarly in five MDR isolates including *E. coli* (1EP, 8EP, 10EP, 11EP, and 27EP); three of them were isolated from a wound of a postoperative infection and two from urinary tract infection (UTI) patients. Four isolates of *E. coli* harbored a set of MDR genes cassettes (*mph(A)*, *qacE*, *dfrA17*, *sul1*, *aadA5*), in addition to IS6100, located closely at the same contigs.

Six MGEs were detected in the same cassette adjacent to each other in one MDR water isolate (16EE), containing po111 plasmid flanked by ISKpn19 and IS102 insertion sequences, and two ARGs (*floR* and *qnrS1*) which fell in brackets of ISKpn19 and ISVsa3.

**Figure 4:**
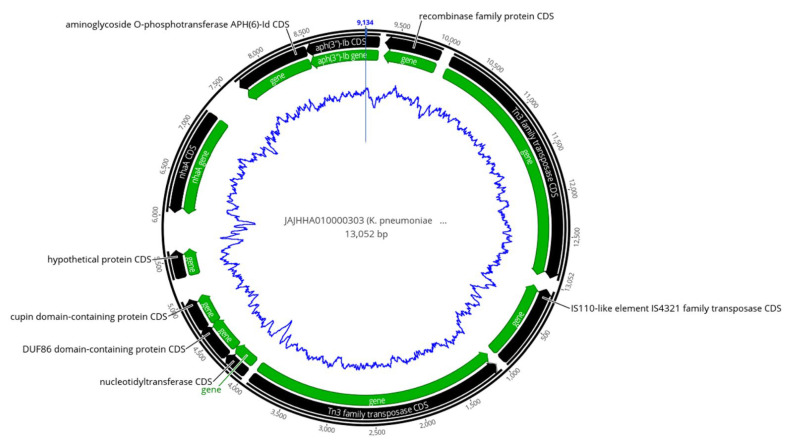
Map of antibiotic resistance and transposases cassette, identified in contig 303 of environmental *K. pneumoniae* (12KE), showing the aminoglycoside-resistant genes flanked by three transposases genes. The outer black circle indicates the length of the contig, black arrows indicate coding sequences (CDS), green arrows indicate genes, and the inner blue zigzag circle indicates GC content.

A *K. pneumoniae* fruit isolate (12KE) showed the presence of aminoglycoside-resistant genes (*aph(3*″*)-Ib, aph(6)-Id*) and the chromosomally mediated *nhaA* gene flanked by three transposases genes (two Tn3 and one 1S110) (Table S5 and Figure 4).

**Figure 5:**
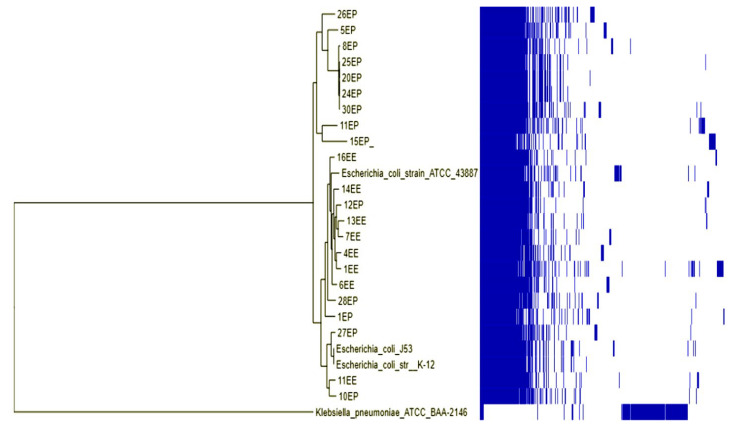
Phylogenomic tree for the clinical and environmental isolates of *E. coli* from different sources in Khartoum and reference strains (*Escherichia coli* J53, K-12, and ATCC_43887), Sudan. Environmental isolates were EE while clinical isolates were EP. The blue blocks indicate gene presence and absence. The *Klebsiella pneumoniae* ATCC_BAA-2146 was used as an outgroup for rooting the tree.

Two MDR isolates of *E. coli* (20EP and 25EP) were isolated from the urine of patients with chronic kidney disease, characterized by the presence of *rmtB* and *bla*_TEM-1B,_ and were harbored in Tn2 transposon. These two isolates were located in the same clade as shown in the phylogenetic tree (Figure 5). Three environmental isolates of *K. pneumoniae* (15KE, 16KE, and 20KE) showed the co-existence of insertion sequences (ISKpn14, ISKpn41, and ISEhe3) and plasmids (IncHI1B, IncFIB(Mar), and repA) in the same contigs (Table S5).

### 2.7. Phylogenetic Analysis

**Figure 6:**
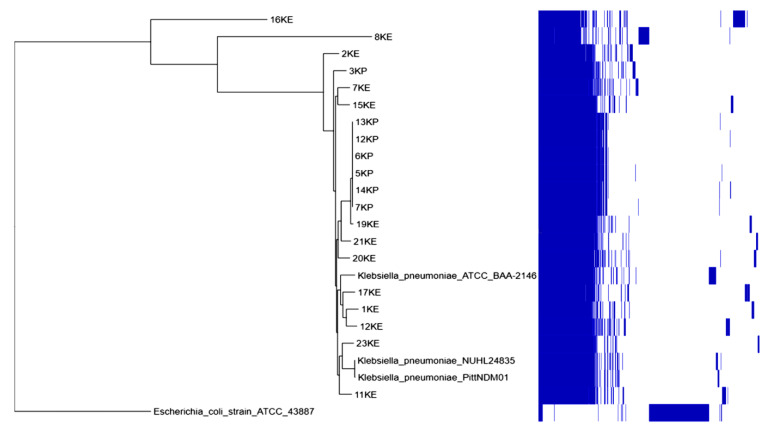
Phylogenomic tree for the clinical and environmental isolates of *K. pneumoniae* from different sources in Khartoum and reference strains (*K. pneumoniae* NUHL24835, PittNDM01, and ATCC_BAA-2146), Sudan. Environmental isolates were KE, while clinical isolates were KP. The blue blocks indicate gene presence and absence, *Escherichia coli* strain ATCC_43887 was used as an outgroup for rooting the tree.

The phylogenetic tree and metadata revealed that most isolates were clustered according to the source of the isolate (environmental or clinical), while few were mixed. Isolate 13EE from the environment and isolate 28EP from the clinical source clustered together. Water isolates (1EE, 4EE, 6EE, 7EE, 14EE, 13EE, and 16EE) and three clinical isolates (8EP, 12EP, and 28EP) were clustered together. Isolates 1KE and 11EE clustered with isolates 10EP and 27EP from patients with a wound infection and UTI, respectively. Isolates 12KE from fruits and 15KE from the table surface were closely related to isolate (13KP) from patients with septicemia; isolates 12KE and 13KE belong to ST45. The four clinical isolates (5KP, 6KP, 7KP, 12KP, 13KP, and 14KP) were closely related to isolate 19KE from fruits, and all belong to ST45 (Figure 5 and Figure 6).

## 3. Discussion

*K. pneumoniae* and *E. coli* have been associated with epidemic and endemic nosocomial infections caused by multidrug infections, mainly ESBL-producing bacteria in Sudan and worldwide [2,10,29]. β-lactamases-producing *K. pneumoniae* and *E. coli*, especially TEM, CTX-M, and SHV type, are the most prevalent species that have spread globally within the hospital and environment [30,31,32,33,34]. ESBL-producing bacteria spread through consumption or cross-contamination. Environmental exposure produces considerably high ESBL-positive *E. coli* levels in vegetables and foods due to insufficient hygiene in irrigation water systems [8,34,35]. In this study, all *K. pneumoniae* and a few numbers of *E. coli* isolated from water and vegetables were ESBL producers, which gives evidence of the spreading of ESBL genes in our environment. This could be as a result of the strong selection pressure exerted by the indiscriminate use of beta-lactam antibiotics in our community [36].

*bla_CTX-M-15_* was documented recently as the most prevalent ESBL gene in Sudan, which was reported in environmental and clinical samples in previous studies [26,27]. This study is in line with these studies, in which we detected the *bla_CTX-M-15_* gene bracketed by ISEc9 and Tn3 transposases in six clinical and one environmental isolate. These transposases play a crucial role in gene transfer and could be one of the reasons behind horizontal gene transfers [37]. Similar to our findings, Madni et al. [14] recently reported *K. pneumoniae* with *bla_CTX-M-15_* bracketed by ISEc9 and Tn3 from South African patients. Four of *bla_CTX-M-15_*, ISEc9-, and Tn3-positive isolates were from neonatal sepsis in the pediatric ward at Soba University Hospital caused by *K. pneumoniae* ST45, suggesting a hospital-acquired infection which is more common in Sudan hospitals [10,38]. Similarly, the *mph(A), qacE*, *dfrA17*, *sul1*, and *aadA5* ARGs have co-existed closely with IS6100 *transposase* in five MDR *E. coli* isolates, three being from a wound of a postoperative infection. A similar set of genes cluster with transposons in MDR *E. coli* was reported by Roy Chowdhury et al. [39] from Australian patients with UTIs.

*bla_CTX-M-15_*-positive isolates were phenotypically resistant to cephalosporin; this finding is expected because this gene was documented with a high affinity to hydrolyze cephalosporin [13]. Here, the *bla_CTX-M-15_* was detected among 15 isolates from clinical and environmental isolates, which is in agreement with a study conducted in Khartoum, which revealed the *bla_CTX-M-15_* gene in isolates recovered from diverse non-clinical niches and belonging to different Enterobacteriaceae species [40,41]. CTX-M-15 β-lactamases are mainly encoded in IncFII plasmids, the host plasmid of the high-risk clone *K. pneumoniae*, and play an important role in its international dissemination [42]. Moreover, different studies from Tanzania, Nigeria, and Tunisia reported the presence of *bla_CTX-M-15_* in IncF-type plasmids from clinical and environmental isolates [33,43].

*E. coli* ST38 is an international high-risk clone responsible for the spreading of the *OXA-48* gene [44]. In this study, ST38 was detected in one MDR *E. coli* isolate from cerebrospinal fluid (CSF), and this isolate was positive for *bla*_CTX-M-15_ and *bla*_TEM-35_.

In this study, we documented one *E. coli* strain (ST120) containing *bla*_DHA-1_ and *qnrB4* associates with four plasmids: IncFIA, IncFIB (AP001918), IncFIC(FII), and IncI1-I(Alpha). The co-occurrence of *bla*_DHA-1_ and *qnrB4* with IncL/M and IncR plasmids has been reported in Europe and Asia, in *Serratia marcescens*, *E. coli*, *K. pneumoniae* (ST11 and ST37), and *Enterobacter cloacae* ST528 [45,46,47]. Additionally, *bla*_NDM-5_ was found on three *E. coli* ST405 isolates in association with IncFII-I ncFIB[*p*B171] plasmid replicons, similar to a study from Japan [48].

*E. coli* and *K. pneumoniae* have the potential to increase the widespread transmission of ARGs via MGEs through the processes of natural competence, transformation, and plasmid trans conjugation that can occur in any environment [8]. There are many plasmids associated with antimicrobial resistance genes in the Enterobacterial group (e.g., *IncF*, *IncA/C*, *IncH*, *IncP*, *IncL/M*, or *IncX*, etc.) [15]. Some of these plasmids, such as *IncF*, are encoded with specific resistance genes such as ESBLs, carbapenems, aminoglycosides, or fluoroquinolones, while IncI2, IncX4, and IncP plasmids are associated with resistance to colistin encoded by the *mcr-1* gene; IncHI1 and IncHI2 plasmids are reported to be associated with MDR [15]. IncI1-Ic and IncFIA-FIB plasmid types were reported in co-existence with multiple types of ESBL-encoding genes (*bla*_CTX-M-3_, *bla*_TEM-1_, and *bla*_SHV-12_) in *K. pneumoniae* from North India [49]. This is similar to our finding in which multiple ESBL-encoding genes (*bla*_OXA-1_, *bla*_CTX-M-15_, *bla*_SHV-26_, *bla*_SHV-11_, and *bla*_SHV-38_), (*bla*_NDM-5_, *bla*_CTX-M-15_, and *bla*_TEM-1B_) and (*bla*_NDM-5_, *bla*_CTX-M-15_, and *bla*_OXA-1_) have been documented with IncI1-Ic and IncFIA-FIB.

The fosfomycin-resistant genes (*fosA* and *fosA5*) were detected as co-resistance in ESBL-producing *K pneumoniae*, this finding is similar to many studies reporting co-resistance of fosfomycin and ESBL in plasmid-mediated resistance [50,51,52].

The genes encoding for sulfonamide-resistant dihydropteroate synthase (*Sul2*), which is consistently co-carried with aminoglycoside-resistant genes (*aph(6)-Id* and *aph(3″)-Ib*) [13], were detected in this study, bracketed by two transposase (IS5075 and IS91) in 5 *K. pneumoniae* isolates. These transposases could be the vehicle for the horizontal gene transfer and dissemination of these genes in our isolates [53]. Interestingly, we reported six MGEs clustered together in one MDR *E. coli* (16EE) from water, which contained a cluster of *sul2*, *dfrA14*, and *tet(A)* genes, and a po111 plasmid flanked by ISKpn19 and IS102 insertion sequences, and two ARGs: the quinolone resistance determinant (*qnrS1*) and florfenicol resistance gene (*floR*); these fall in the brackets of ISKpn19 and ISVsa3. The presence of ARGs between transposable elements will help in the easy transposition of genes and can mediate their mobility between drug-sensitive organisms [54]. In later studies from the Khartoum locality, they noted an increase in the presence of ARGs in *E. coli* isolated from drinking water [40,41].

Future studies from different geographical regions in Sudan with large sample sizes should be considered to better understand the possible role of the environment in the dissemination of ARGs.

## 4. Materials and Methods

### 4.1. Bacterial Strains and Antimicrobial Susceptibility Testing

A total of 42 consecutive isolates were collected randomly between March and July 2021 from hospitalized patients in Soba University Hospital (*n* = 20) and the environment (*n* = 22). The clinical isolates were collected at the clinical microbiology laboratory of Soba University Hospital as part of their routine clinical procedure; the isolates were from various samples, including blood, urine, pus, wounds, cerebrospinal fluid, and catheter tips. The environmental isolates were from swabs obtained from vegetables, markets, and water stations (Table 1). Environmental samples were collected and processed according to published protocols [40,55]. Isolates were primarily identified using Gram staining and standard biochemical tests, which include citrate utilization test, glucose and lactose fermentation in Kligler iron agar tubes, urease and indole [56], and according to their colors and growth characteristics on chromogenic media. Phenotypic antimicrobial susceptibility testing (AST) was performed using the disk diffusion method against an antibiotic panel including amoxicillin-clavulanate (30 µg), cefuroxime (30 µg), ceftriaxone (30 µg), ceftazidime (30 µg), cephalexin (30 µg), meropenem (10 µg), imipenem (10 µg), amikacin (30 µg), gentamicin (10 µg), ciprofloxacin (5 µg), trimethoprim-sulfamethoxazole (25 µg), and nitrofurantoin (300 μg). American Type Culture Collection (ATCC) strains including *P. aeruginosa* ATCC 27853 and *E. coli* ATCC 25922 were used as quality controls. Results were interpreted according to the Clinical and Laboratory Standards Institute (CLSI) guidelines [57].

### 4.2. Whole-Genome Sequencing and Molecular Analysis

Genomic DNA was extracted from overnight bacterial growth using the Guanidine hydrochloride method as described by Sabeel et al. [58]. The integrity and quantity of extracted DNA was estimated by gel electrophoresis and Nanodrop, Qubit (Thermo Scientific, Waltham, MA, USA).

WGS was performed by Novogene Company (China) using an Illumina HiSeq 2500 platform (Illumina, San Diego, CA, USA), 2 × 150 bp paired-end reads were generated with 100 X coverage. Trimmomatic 0.36 [59] was used to remove low-quality reads, adapters, and reads containing *n* > 10%. ContEst16S was used to screen assembled genomes for contamination by either cells or DNAs from other organisms. De novo assembly was conducted by Velvet v1.2.10 [60] and PATRIC (Pathosystems Resource Integration Center) server. The assembled bacterial genomes were identified in species, strain levels, and STs using MLST 2.0 and PubMLST [61] databases. The novel sequence types (ST) were assigned by the Pasteur MLST database. Genome annotation was achieved by the RAST server [62] and NCBI Prokaryotic Genome Annotation Pipeline (PGAP) [63]. Antimicrobial resistomes were predicted using Resistance Gene Identifier (RGI) and ResFinder [64]. Virulence genes were also investigated using VirulenceFinder 2.0 (Center for Genomic Epidemiology, DTU, Lyngby, Denmark) and Mobile Element Finder [65]. Plasmids, insertion sequences and transposons, and Human pathogen probability were predicted by Plasmid Finder 2.1, IS Finder, and Pathogen Finder [66], respectively. Resistant genes and MGEs maps were visualized by Geneious Prime 2021 trial version. The assembled contigs were submitted to GenBank under the Bioproject PRJNA767482.

### 4.3. Phylogenetic Analysis

The phylogenetic tree for *E. coli* and *K. pneumoniae* was constructed via tools *available through* the Galaxy platform [67], the assembled contigs were annotated using Prokka [68], and the generated GFF3 format was used as input for Roary [69] with a default minimum of 95% identity for BLASTp to estimate the pan-genome. The aligned core genes generated from Roary were used as input for RaXML [70] for the reconstruction of the phylogenetic tree. The phylogenetic graph was visualized by Phandango using files generated from RAxML (raxml tree) and Roary (gene presence absence cvs).

## 5. Conclusions

This study sheds light on the spread of ARGs and/or MGEs in clinical and environmental isolates of *E. coli* and *K. pneumoniae*. *bla_CTX-M-15_* bracketed between *ISEc9 and Tn3* transposases is disseminated in environmental and clinical isolates. Four *K. pneumoniae* strains possessed *bla_CTX-M-15_* bracketed by ISEc9 and Tn3, *Sul2*, and contained *aph(6)-Id* and *aph(3″)-Ib*) bracketed by IS5075, and IS91 belonging to ST45, which were potentially endemic in the delivery room or pediatrics unit of Soba University Hospital. A similar pattern of the clustering of IS6100 with MDR genes cassettes (*mph(A)*, *qacE*, *dfrA17*, *sul1*, and *aadA5*) was observed in *E. coli* isolated from patients with postoperative wound infections at Soba University Hospital, indicating possible hospital-acquired infections. This finding necessitates a rapid response from stakeholders to initiate a program for infection prevention and control measures to detect such clones disseminated in communities and hospitals.

## Figures and Tables

**Table 1:** Information about the study isolates, source, Specimen type, pubMLST, ST, Human pathogen probability, Accession numbers and AST.

ID	Source	Specimen Type	PubMLST	ST	Accession No.
1EP	Clinical sample	Wound swab	*E. coli*	120	JAJGXP000000000
5EP	Clinical sample	CSF	*E. coli*	38	JAJGXO000000000
8EP	Clinical sample	Urine	*E. coli*	405	JAJHQC000000000
10EP	Clinical sample	Wound swab	*E. coli*	773	JAJGXN000000000
11EP	Clinical sample	Wound swab	*E. coli*	648	JAJGXM000000000
15EP	Clinical sample	Urine	*E. coli*	73	JAJGXK000000000
20EP	Clinical sample	Urine	*E. coli*	405	JAJGXJ000000000
24EP	Clinical sample	Acetic fluid	*E. coli*	405	JAJGXI000000000
25EP	Clinical sample	Urine	*E. coli*	405	JAJGXH000000000
26EP	Clinical sample	Wound Swab	*E. coli*	340	JAJHGQ000000000
27EP	Clinical sample	Urine	*E. coli*	656	JAJGXG000000000
28EP	Clinical sample	Urine	*E. coli*	410	JAJGXF000000000
30EP	Clinical sample	Urine	*E. coli*	405	JAJGXE000000000
1EE	Hands	Swab	*E. coli*	2522	JAJGXX000000000
4EE	Hands	Swab	*E. coli*	58	JAJGXW000000000
6EE	Water	Water sample	*E. coli*	4038	JAJGXV000000000
7EE	Water	Water sample	*E. coli*	448	JAJGXU000000000
11EE	Vegetables	Swab	*E. coli*	522	JAJGXU000000000
12EP	Surface	Swab	*E. coli*	2280	JAJGXL000000000
13EE	Surface	Swab	*E. coli*	1308	JAJGXS000000000
14EE	Water	Water sample	*E. coli*	1508	JAJGXR000000000
16EE	Water	Water sample	*E. coli*	1146	JAJGXQ000000000
1KE	Vegetables	Swab	*K. pneumoniae*	2365	JAJHHI000000000
2KE	Surface	Swab	*K. pneumoniae*	2177	JAJHHH000000000
3KP	Clinical sample	Urine	*K. pneumoniae*	76	JAJHHG000000000
5KP	Clinical sample	Blood	*K. pneumoniae*	45	JAJHHF000000000
6KP	Clinical sample	Blood	*K. pneumoniae*	45	JAJHHE000000000
7KE	Surface	Swab	*K. pneumoniae*	700	JAJHHD000000000
7KP	Clinical sample	Blood	*K. pneumoniae*	45	JAJHHC000000000
8KE	Water	Water sample	*K. pneumoniae*	1584	JAJHNR000000000
11KE	Vegetables	Swab	*K. pneumoniae*	1507	JAJHHB000000000
12KE	Fruits	Swab	*K. pneumoniae*	45	JAJHHA000000000
12KP	Clinical sample	Blood	*K. pneumoniae*	45	JAJHGZ000000000
13KP	Clinical sample	Blood	*K. pneumoniae*	45	JAJHGY000000000
14KP	Clinical sample	Blood	*K. pneumoniae*	45	JAJHGX000000000
16KE	Surface	Swab	*K. pneumoniae*	22,233 *	JAJHNQ000000000
17KE	Vegetables	Swab	*K. pneumoniae*	22,234 *	JAJHGW000000000
19KE	Vegetables	Swab	*K. pneumoniae*	45	JAJHGV000000000
20KE	Vegetables	Swab	*K. pneumoniae*	5624	JAJHGU000000000
15KE	Surface	Swab	*K. pneumoniae*	5808	JAJHGT000000000
21KE	Surface	Swab	*K. pneumoniae*	3335	JAJHGS000000000
23KE	Water	Water samples	*K. pneumoniae*	1504 *	JAJHGR000000000

* Assigned novel ST.

**Table 2:** Presentation of antimicrobial susceptibility profiles.

ID	Phenotypic AST							
	Beta-Lactam					Aminoglycosides and Fluoroquinolones		
	MR	IMP	CAZ	CTR	AMC	AK	GEN	CIP
1EP	S	S	R	R	R	S	S	S
5EP	S	S	R	R	R	S	R	S
8EP	S	S	R	R	R	S	S	R
10EP	S	S	R	R	R	S	S	S
11EP	S	S	R	R	R	R	R	S
12EP	S	S	S	S	R	I	I	S
15EP	S	S	S	S	R	S	S	S
20EP	S	S	R	R	R	R	R	R
24EP	S	S	S	S	R	S	S	S
25EP	S	S	R	R	R	S	S	R
26EP	S	S	R	R	R	S	S	R
27EP	S	S	R	R	R	S	S	S
28EP	S	S	R	R	R	S	S	S
30EP	S	S	R	R	R	R	R	R
1EE	S	S	S	S	S	S	S	S
4EE	S	S	S	S	S	S	S	S
6EE	S	S	S	S	S	S	S	S
7EE	S	S	S	R	R	S	S	S
11EE	S	S	S	S	R	S	R	S
13EE	S	S	S	S	R	S	S	S
14EE	S	S	S	S	R	I	I	S
16EE	S	S	S	S	R	S	R	R
3KP	S	S	S	S	R	S	S	S
5KP	S	S	R	R	R	S	R	S
6KP	S	S	R	R	R	S	R	R
7KP	S	S	R	R	R	S	R	R
12KP	S	S	R	R	R	S	S	R
13KP	S	S	R	R	R	S	S	S
14KP	S	S	R	R	R	S	S	S
1KE	S	S	S	S	R	S	S	S
2KE	S	S	S	S	R	S	S	S
7KE	S	S	S	S	R	S	S	S
8KE	S	S	S	S	R	S	S	S
11KE	S	S	S	S	R	S	S	S
12KE	S	S	S	S	R	S	S	S
16KE	S	S	S	S	R	S	S	S
15KE	S	S	S	S	R	S	S	S
17KE	S	S	S	S	R	S	S	S
19KE	S	S	R	R	R	S	S	S
20KE	S	S	S	S	R	S	S	S
21KE	S	S	S	S	R	S	S	S
23KE	S	S	S	S	R	S	S	S

Abbreviations: ST, strain; S, sensitive; R, resistant; I, Intermediate; -, negative; MR, meropenem; IMP, imipenem; CAZ, cefatzidime; CTX, cefotaxime; GEN, gentamicin; CTR, Co-trimoxazole; CIP, ciprofloxacin; AK, amikacin; AMC, amoxicillin-clavulanic acid.

**Table 3:** Antimicrobial-resistance genes identified in the isolates, red color means detected.

ID	Beta Lactam																				Aminoglycosides and Fluoroquinolones															Others											
	*bla_CTX-M-15_*	*bla_CTX-M-216_*	*bla_OXA-1_*	*bla_DHA-1_*	*bla_TEM-35_*	*bla_TEM-1_*	*bla_CMY-141_*	*bla_CMY-42_*	*bla_NDM-5_*	*bla_SHV-1_*	*bla_SHV-12_*	*bla_SHV-11_*	*bla_SHV-26_*	*bla_SHV-38_*	*bla_SHV-71_*	*bla_LEN16_*	*ompK37*	*ompK36*	*bla_LEN16_*	*blaEC*	*qnrB4*	*aac(6′)-Ib-cr*	*aac(3)-IIa*	*aadA5*	*aph(3″)-Ib*	*aph(6)-Id*	*mdf(A)*	*Mdf*	*rmtB*	*qepA4*	*qacE*	*qnrS1*	*OqxB*	*OqxA*	*acrR*	*sul2*	*Sul1*	*dfrA*	*mph(A)*	*catB3*	*catA1*	*tet(A)*	*tet(B)*	*tet(39)*	*sitABCD*	*fosA*	*erm(C)*
1EP																																															
5EP																																															
8EP																																															
10EP																																															
11EP																																															
12EP																																															
15EP																																															
20EP																																															
24EP																																															
25EP																																															
26EP																																															
27EP																																															
28EP																																															
30EP																																															
1EE																																															
4EE																																															
6EE																																															
7EE																																															
11EE																																															
13EE																																															
14EE																																															
16EE																																															
1KE																																															
2KE																																															
3KP																																															
5KP																																															
6KP																																															
7KE																																															
7KP																																															
8KE																																															
11KE																																															
12KE																																															
12KP																																															
13KP																																															
14KP																																															
15KE																																															
16KE																																															
17KE																																															
19KE																																															
20KE																																															
21KE																																															
23KE																																															

**Table 4:** Types and distributions of plasmids between studied isolates. Blue color means detected.

ID	IncFIA	IncFIB	IncFII	IncI1-I(Alpha)	IncY	IncR	IncFIB(H89-PhagePlasmid)	IncFIB(pB171)	IncFIB(pKPHS1)	IncFII(pHN7A8)	IncFIC(FII)	ncFII(pRSB107)	IncFIB(AP001918)	Col156	Col(BS512)	Col440I	IncI(Gamma)	IncFII(pAMA1167-NDM-5)	IncFIB(pNDM-Mar)	IncHI1B(pNDM-MAR)	IncFII(pCoo)	IncFIA(HI1)	p0111	IncFII(29)	IncFIB(K)(pCAV1099-114)	FIA(pBK30683)	IncFIB(K)	IncFII(K)	IncFII(pKP91)	ColpVC	pKP1433
1EP																															
5EP																															
8EP																															
10EP																															
11EP																															
12EP																															
15EP																															
20EP																															
24EP																															
25EP																															
26EP																															
27EP																															
28EP																															
30EP																															
1EE																															
4EE																															
6EE																															
7EE																															
11EE																															
13EE																															
14EE																															
16EE																															
1KE																															
2KE																															
3KP																															
5KP																															
6KP																															
7KE																															
7KP																															
8KE																															
11KE																															
12KE																															
12KP																															
13KP																															
14KP																															
16KE																															
17KE																															
19KE																															
20KE																															
15KE																															
21KE																															
23KE																															

## Data Availability

The data of this project were submitted to GenBank under the Bioproject PRJNA767482, and in the additional files.

## Supplementary Materials

The following supporting information can be downloaded at: https://www.mdpi.com/article/10.3390/ph15081011/s1. Table S1: Genomic features of *E. coli* and *K. pneumonia* isolates; Table S2: Point mutations associated with antibiotic resistance; Table S3: Genes associated with antibiotic efflux, antibiotic target alteration and protection; Table S4: Virulence and transposases genes detected in *K. pneumonia* and *E. coli* isolates; Table S5: Co-occurrence of antimicrobial-resistance genes and transposases; Figure S1: Map of different antibiotic-resistant genes in clinical *K. pneumonia* (5KP); Map of different antibiotic resistant genes, transposases, and plasmid, clustered in clinical *K. pneumonia* (7KP); Figure S3: Map of different antibiotic resistant genes, transposases, and plasmid, clustered in clinical *K. pneumonia* (6KP); Figure S4: Map of different antibiotic resistant genes, transposases, and plasmid, clustered in clinical *K. pneumonia* (13KP) isolate; Figure S5. Map of different antibiotic resistant genes, transposases, and plasmid, clustered in contig 156 of clinical *K. pneumonia* (14KP) isolate.
