# Supplementary material for: Co-Occurrence of β-Lactam and Aminoglycoside Resistance Determinants among Clinical and Environmental Isolates of Klebsiella pneumoniae and Escherichia coli: A Genomic Approach
Source: Pharmaceuticals (Basel). 2022 Aug 17;15(8):1011. doi: 10.3390/ph15081011 (PMC9416466; doi:10.3390/ph15081011)
Supplement: Supplementary file 1 [file pharmaceuticals-15-01011-s001.zip › pharmaceuticals-1798399-supplementary.pdf]

Supplementary

Table S1. Genomic features of *E. coli* and *K. pneumonia* isolates

ID	PubMLST	Biosample	Contigs	Genome Length	GC Content	CDS	tRNA	rRNA	N50	Median coverage depth
1EE	*E. coli*	SAMN22568277	897	5475233	50.47	6032	67	2	19330	218
4EE	*E. coli*	SAMN22568278	550	5140546	50.39	5302	34	5	28232	223
6EE	*E. coli*	SAMN22568279	102	4933387	50.95	4873	38	8	185511	223
7EE	*E. coli*	SAMN22568280	118	4813344	50.73	4762	75	3	151269	272
11EE	*E. coli*	SAMN22568280	322	4738655	50.7	4800	40	5	40928	202
13EE	*E. coli*	SAMN22568282	256	4582345	50.9	2326	21	2	56438	185
14EE	*E. coli*	SAMN22568283	323	4888694	50.67	4143	34	4	195936	239
16EE	*E. coli*	SAMN22568284	100	4856794	50.69	4783	77	4	162048	308
1EP	*E. coli*	SAMN22568285	891	4954601	50.84	5413	39	4	28399	147
5EP	*E. coli*	SAMN22568286	186	5350986	50.45	5358	87	5	150097	185
8EP	*E. coli*	SAMN22568287	645	5273033	50.56	5475	46	5	105067	138
10EP	*E. coli*	SAMN22568288	193	4767339	50.83	4798	76	2	78177	238
11EP	*E. coli*	SAMN22568289	633	5425295	50.37	5728	46	3	70463	133
12EP	*E. coli*	SAMN22568290	308	4662795	50.79	4651	41	5	238555	293
15EP	*E. coli*	SAMN22568291	87	4989418	50.48	4895	79	4	331963	292
20EP	*E. coli*	SAMN22568292	603	5161678	50.68	5357	43	5	44314	156
24EP	*E. coli*	SAMN22568293	711	5211395	50.6	5490	44	4	53114	88
25EP	*E. coli*	SAMN22568294	631	5159300	50.68	5373	43	4	41360	148
26EP	*E. coli*	SAMN22860965	139	5182400	50.55	5222	86	4	163013	326
27EP	*E. coli*	SAMN22568295	486	4951868	50.69	5083	44	7	41947	135
28EP	*E. coli*	SAMN22568296	367	4930045	50.58	4994	45	4	46893	126
30EP	*E. coli*	SAMN22568297	149	5227267	50.6	2960	41	2	105971	241
1KE	*K. pneumoniae*	SAMN22857709	64	5415845	57.08	5337	75	3	264164	269
2KE	*K. pneumoniae*	SAMN22857710	307	5287854	57.24	5340	37	6	38144	197
7KE	*K. pneumoniae*	SAMN22857714	379	5357423	57.27	5468	40	5	58874	169
8KE	*K. pneumoniae*	SAMN22857716	263	5258392	57.88	5231	38	4	50455	190
11KE	*K. pneumoniae*	SAMN22857717	66	5404444	57.27	4460	63	2	236656	247
12KE	*K. pneumoniae*	SAMN22857718	540	5762894	56.77	5941	39	7	38802	166

15KE	*K. pneumoniae*	SAMN22857726	91	5488393	56.99	5405	75	3	350278	311
16KE	*K. pneumoniae*	SAMN22857722	44	5671546b	56.96	5569	76	5	451883	292
17KE	*K. pneumoniae*	SAMN22857723	156	5415843	57.22	5342	76	4	125571	369
19KE	*K. pneumoniae*	SAMN22857724	65	5458400	57.16	5353	75	5	357556	321
20KE	*K. pneumoniae*	SAMN22857725	108	5310880	57.4	5214	76	4	189136	540
21KE	*K. pneumoniae*	SAMN22857727	21	5294801	57.43	5148	76	5	272625	290
23KE	*K. pneumoniae*	SAMN22857728	31	5211037	57.48	5052	74	4	373869	285
3KP	*K. pneumoniae*	SAMN22857711	360	5454149	57.23	5491	34	6	51712	191
5KP	*K. pneumoniae*	SAMN22857712	387	5535606	57.02	5634	40	5	51948	148
6KP	*K. pneumoniae*	SAMN22857713	386	5535420	57.02	5628	41	5	54815	168
7KP	*K. pneumoniae*	SAMN22857715	391	5534264	57.03	5631	42	6	56535	135
12KP	*K. pneumoniae*	SAMN22857719	465	5603818	56.77	5720	50	10	58941	128
13KP	*K. pneumoniae*	SAMN22857720	379	5535338	57.03	5618	39	4	56449	108
14KP	*K. pneumoniae*	SAMN22857721	494	5535678	57.03	5704	40	4	30293	169

Table S2: Point mutations associated with antibiotic resistance

**Isolate ID**	**Species**	**beta-lactam antibiotics**	**Fosfomycin**			**Pulvomycin**	**MDR**	**fluoroquinolone**		
		**PBP3**	**cyaA**	**UhpT**	**GlpT**	**EF-Tu**	**acrR**	**parC**	**gyrA**	**marR**
1EE	*E. coli*	D350N, S357N	-	-	E448K	R234F	-	-	-	Y137H, G103S
4EE	*E. coli*	D350N, S357N	-	-	E448K	R234F	-	-	-	Y137H, G103S
6EE	*E. coli*	-	-	E350Q	E448K	-	-	-	-	Y137H, G103S
7EE	*E. coli*	-	-	-	E448K	R234F	-	-	-	Y137H, G103S
11EE	*E. coli*	-	-	-	E448K	R234F	-	-	-	Y137H, G103S
13EE	*E. coli*	-	-	-	E448K	R234F	-	-	-	Y137H, G103S
14EE	*E. coli*	-	-	-	-	-	-	-	-	-
16EE	*E. coli*	D350N, S357N	-	-	E448K	-	-	-	-	Y137H, G103S

1EP	*E. coli*	D350N, S357N	-	-	E448K	R234F	-	-	-	Y137H, G103S
5EP	*E. coli*	-	S352T	-	E448K	R234F	-	-	-	Y137H, G103S
8EP	*E. coli*	-	S352T	-	E448K	R234F	-	S80I	D87N, S83L	Y137H, G103S
10EP	*E. coli*	-	S352T	-	E448K	R234F	-	S80I	D87N, S83L	-
11EP	*E. coli*	-	S352T	-	E448K	R234F	-	S80I	D87N, S83L	Y137H, G103S
12EP	*E. coli*	-	-	-	E448K	R234F	-	-	-	Y137H, G103S
15EP	*E. coli*	-	-	-	E448K	R234F	-	-	-	Y137H, G103S
20EP	*E. coli*	-	S352T	-	E448K	R234F	-	S80I	D87N, S83L	-
24EP	*E. coli*	-	S352T	-	E448K	R234F	-	S80I	D87N, S83L	Y137H, G103S
25EP	*E. coli*	-	S352T	-	E448K	R234F	-	S80I	-	-
26EP	*E. coli*	-	-	E350Q	E448K	R234F	-	-	-	Y137H, G103S
27EP	*E. coli*	-	-	-	-	R234F	-		S83L	-
28EP	*E. coli*	-	-	-	E448K	R234F	-	S80I	D87N, S83L	Y137H, G103S
30EP	*E. coli*	-	S352T		E448K	R234F	-	S80I	D87N, S83L	-
1KE	*K. pneumoniae*	-	-	E350Q	-	-	-		-	-
2KE	*K. pneumoniae*	-	-	E350Q	-	R234F	-		-	-
7KE	*K. pneumoniae*	-	-	E350Q	-	R234F	-		-	-
8KE	*K. pneumoniae*	-	-	E350Q	-	-	Y114F, V165I	-	-	-
11KE	*K. pneumoniae*	-	-	E350Q	-	R234F	-		-	-
12KE	*K. pneumoniae*	-	-	E350Q	-	R234F	-		-	-
15KE	*K. pneumoniae*	-	-	E350Q	-	R234F	-		-	-
16KE	*K. pneumoniae*	-	-	E350Q	-	-	-	-	-	-
17KE	*K. pneumoniae*	-	-	E350Q	-	-	-	-	-	-
19KE	*K. pneumoniae*	-	-	nil	-	-	-	-	-	-

20KE	*K. pneumoniae*	-	-	nil	-	-	-	-	-	-
21KE	*K. pneumoniae*	-	-	nil	-	-	-	-	-	-
23KE	*K. pneumoniae*	-	-	E350Q	-	-	-	-	-	-
3KP	*K. pneumoniae*	-	-	E350Q	-	-	-	-	-	-
5KP	*K. pneumoniae*	-	-	E350Q	-	-	-	-	-	-
6KP	*K. pneumoniae*	-	-	E350Q	-	-	-	-	-	-
7KP	*K. pneumoniae*	-	-	E350Q	-	-	-	-	-	-
12KP	*K. pneumoniae*	-	-	E350Q	-	-	-	-	-	-
13KP	*K. pneumoniae*	-	-	E350Q	-	-	-	-	-	-
14KP	*K. pneumoniae*	-	-	E350Q	-	-	-	-	-	-

Table S3. **Genes associated with antibiotic efflux, antibiotic target alteration and protection**

ID	Species	Source	Gene
1EE	*E. coli*	Community	acrB, msbA, emrR, emrB, mdtG, AcrE, cpxA, H-NS, marA, evgA, mdtE, TolC, sul2, emrA, CRP, bacA, mdtH, AcrF, AcrS
4EE	*E. coli*	Community	mdtG, mdtH, marA, msbA, acrB, E. coli acrA, AcrE, E. coli ampH beta-lactamase, evgA, cpxA, emrB, emrR, TolC, mdtE, H-NS, baeR, E. coli emrE
6EE	*E. coli*	Community	AcrE, TolC, H-NS, mdtG, baeS, acrB, E. coli acrA, mdtE, msbA, emrR, emrB, evgA, cpxA, marA
7EE	*E. coli*	Community	evgA, baeR, baeS, mdtG, mdtH, H-NS, marA, acrB, E. coli acrA, emrR, TolC, cpxA, msbA, mdtE, AcrE
11EE	*E. coli*	Community	E. coli acrA, H-NS, bacA, msbA, mdtG, mdtH, AcrS, AcrE, mdtC, YojI, PmrF, emrB, mdtN, TolC, gadX, mdtF, mdtE, evgA, emrK, marA
13EE	*E. coli*	Community	acrB, E. coli acrA, mdtH, mdtG, emrY, evgA, msbA, PmrF, TolC, cpxA, emrB, AcrE, AcrS, mdtE, H-NS, baeS, marA
14EE	*E. coli*	Community	baeS, PmrF, evgA, TolC, AcrE, mdtG, H-NS, emrR, emrB, mdtE, E. coli acrA, acrB, E. coli ampH beta-lactamase, msbA, cpxA, marA

16EE	*E. coli*	Community	baeS, PmrF, H-NS, msbA, emrY, evgA, AcrE, E. coli acrA, acrB, TolC, cpxA, marA, emrB, emrR
1EP	*E. coli*	Clinical	marA, acrA, acrB, evgA, QnrB4, DHA-1, sul1, mphA, H-NS, cpxA, msbA, mdtG, mdtH, AcrE, ampH, emrR, PmrF, mdtE, bacA, mdtP, mdtO, mdtN, baeRbaeS
5EP	*E. coli*	Clinical	TolC, baeR, marA, msbA, emrB, emrR, evgA, emrK, mdtH, mdtG, mdtF, H-NS, cpxA
8EP	*E. coli*	Clinical	mdtH, TolC, marA, evgA, emrK, emrY, baeR, msbA, emrR, emrB, H-NS, sul2, dfrA17, qacEdelta1, sul1
10EP	*E. coli*	Clinical	mdtG, mdtH, H-NS, msbA, acrD, E. coli acrA, acrB, evgS, evgA, emrK, PmrF, YojI, bacA, TolC, mdtP, mdtN, marA, emrR, emrB, mdtE, gadX, dfrA17, qacEdelta1
11EP	*E. coli*	Clinical	mdtG, marA, cpxA, TolC, emrY, evgA, msbA, acrB, E. coli ampH beta-lactamase, kdpE, baeR, mphA, sul1, qacEdelta1, aadA5, dfrA17, H-NS
12EP	*E. coli*	Clinical	baeS, PmrF, emrY, evgA, mdtG, H-NS, emrR, emrB, marA, AcrE, TolC, msbA, mdtE, E. coli acrA, cpxA
15EP	*E. coli*	Clinical	evgA, baeR, emrR, H-NS, gadW, cpxA, mdtH
20EP	*E. coli*	Clinical	emrK, emrY, H-NS, mdtG, mdtH, baeR, emrR, TolC, sul2, evgA, rmtB, marA, msbA, catI, QepA4, sul1, qacEdelta1, dfrA12, emrB
24EP	*E. coli*	Clinical	baeR, H-NS, mdtH, mdtG,TolC, marA, emrY, emrK, evgA, sul2, emrR, msbA, emrB, QepA4, sul1, qacEdelta1, dfrA12, rmtB
25EP	*E. coli*	Clinical	marA, emrK, evgA, mdtG, mdtH, msbA, H-NS, emrR, dfrA12, qacEdelta1, sul1, BRP(MBL), emrB, TolC, baeR, sul2, rmtB, catI, QepA4
26EP	*E. coli*	Clinical	emrB, emrR, mdtH, cpxA, H-NS, marA, evgA, emrK, emrY, sul2
27EP	*E. coli*	Clinical	E. coli acrA, acrB, E. coli ampH beta-lactamase, YojI, PmrF, evgA, kdpE, TolC, msbA, baeS, mdtG, mdtP, mdtN, AcrS, AcrE, emrA, emrB, H-NS, mdtF, gadX, marA, cpxA, bacA, mdtH
28EP	*E. coli*	Clinical	mdtG, evgA, AcrE, AcrS, emrR, msbA, TolC, marA, E. coli acrA, acrB, mdtB, cpxA, mdtE, H-NS,
30EP	*E. coli*	Clinical	evgA, emrK, emrY, TolC, msbA, emrB, emrR, baeR, mdtG, mdtH, H-NS, marA
1KE	*K. pneumoniae*	Community	LptD, oqxA, K. pneumoniae KpnF, QnrS1, K. pneumoniae KpnH, K. pneumoniae KpnG, emrR, OmpA, CRP, ArnT, eptB, K. pneumoniae OmpK37
2KE	*K. pneumoniae*	Community	K. pneumoniae KpnF, marA, adeF, LptD, H-NS, msbA, OmpA, ArnT, CRP, oqxA, adeF, emrR, K. pneumoniae KpnG, K. pneumoniae KpnH, K. pneumoniae KpnE, K. pneumoniae OmpK37, eptB, baeR
7KE	*K. pneumoniae*	Community	K. pneumoniae KpnF, LptD, K. pneumoniae KpnE, K. pneumoniae OmpK37, K. pneumoniae KpnH, K. pneumoniae KpnG, emrR, adeF, oqxA, CRP, msbA, baeR, adeF, H-NS, ArnT, marA, tet(A),
8KE	*K. pneumoniae*	Community	K. pneumoniae OmpK37, oqxA, adeF, emrR, K. pneumoniae KpnG, K. pneumoniae KpnH, H-NS, LptD, eptB, CRP, marA, K. pneumoniae KpnF, K. pneumoniae KpnE, msbA, OmpA, adeF, ArnT,
11KE	*K. pneumoniae*	Community	oqxA, LptD, K. pneumoniae KpnF, marA, emrR, K. pneumoniae KpnG, K. pneumoniae KpnH, adeF, eptB, ArnT, CRP, baeR, msbA, OmpA, K. pneumoniae OmpK37, H-NS, K. pneumoniae KpnE
12KE	*K. pneumoniae*	Community	LptD, oqxA, K. pneumoniae KpnF, K. pneumoniae OmpK37, marA, ArnT, OmpA, msbA, eptB, CRP, adeF, baeR, K. pneumoniae KpnH, K. pneumoniae KpnG, emrR, H-NS

15KE	*K. pneumoniae*	Community	LptD, K. pneumoniae KpnF, baeR, H-NS, K. pneumoniae Kpn, H, K. pneumoniae KpnG, emrR, oqxA, adeF , OmpA, msbA, K. pneumoniae KpnE, K. pneumoniae OmpK37, ArnT, CRP, eptB, marA
16KE	*K. pneumoniae*	Community	K. pneumoniae OmpK37, K. pneumoniae KpnE, K. pneumoniae KpnF, marA, H-NS, msbA, baeR, adeF, K. pneumoniae KpnH, K. pneumoniae KpnG, emrR, oqxA, LptD, eptB, ArnT, CRP
17KE	*K. pneumoniae*	Community	K. pneumoniae KpnE, K. pneumoniae KpnF, H-NS, msbA, OmpA, ArnT, eptB, K. pneumoniae KpnG, emrR, adeF, oqxA, baeR, K. pneumoniae OmpK37, CRP, marA, LptD
19KE	*K. pneumoniae*	Community	LptD, K. pneumoniae KpnF, baeR, H-NS, marA, K. pneumoniae KpnG, ArnT, CRP, K. pneumoniae KpnE, eptB
20KE	*K. pneumoniae*	Community	K. pneumoniae KpnF, LptD, K. pneumoniae KpnE, eptB, ArnT, CRP, OmpA, oqxA, K. pneumoniae KpnG, K. pneumoniae KpnH
21KE	*K. pneumoniae*	Community	K. pneumoniae KpnF, LptD, oqxA, K. pneumoniae KpnG, K. pneumoniae KpnE, K. pneumoniae OmpK37, H-NS, CRP, ArnT
23KE	*K. pneumoniae*	Community	LptD, K. pneumoniae KpnF, K. pneumoniae KpnE, K. pneumoniae OmpK37, K. pneumoniae KpnG, oqxA, CRP, ArnT
3KP	*K. pneumoniae*	Clinical	oqxA, LptD, K. pneumoniae KpnF, K. pneumoniae KpnG, eptB, OmpA, CRP, ArnT, K. pneumoniae KpnE, K . pneumoniae OmpK37
5KP	*K. pneumoniae*	Clinical	QnrB1, LptD, dfrA14, K. pneumoniae KpnF, sul2, CRP, eptB, oqxA, adeF, emrR, K. pneumoniae KpnG, K. pneumoniae KpnH, OmpA, msbA, ArnT, baeR
6KP	*K. pneumoniae*	Clinical	LptD, K. pneumoniae KpnF, dfrA14, sul2, QnrB1, ArnT, H-NS, OmpA, msbA, marA, baeR, K. pneumoniae KpnE, CRP, K. pneumoniae KpnH, K. pneumoniae KpnG, oqxA, K. pneumoniae OmpK37
7KP	*K. pneumoniae*	Clinical	K. pneumoniae KpnF, LptD, dfrA14, QnrB1, oqxA, adeF, emrR, K. pneumoniae KpnG, K. pneumoniae KpnH, baeR, ArnT, H-NS, K. pneumoniae KpnE, marA, CRP, eptB, OmpA, K. pneumoniae OmpK37
12KP	*K. pneumoniae*	Clinical	LptD, K. pneumoniae KpnF, sul2, QnrB1, dfrA14, CRP, ArnT, eptB, oqxA, K. pneumoniae KpnG, OmpA, K . pneumoniae KpnE, H-NS, K. pneumoniae OmpK37
13KP	*K. pneumoniae*	Clinical	QnrB1, LptD, K. pneumoniae KpnF, dfrA14, sul2, msbA, oqxA, adeF, emrR, K. pneumoniae KpnG, K. pneumoniae KpnH, marA, baeR, K. pneumoniae OmpK37, OmpA, eptB, ArnT, K. pneumoniae KpnE, H-NS, CRP
14KP	*K. pneumoniae*	Clinical	LptD, QnrB1, sul2, dfrA14, K. pneumoniae KpnF, emrR, K. pneumoniae KpnG, K. pneumoniae KpnH, eptB, K. pneumoniae OmpK37, marA, OmpA, msbA, CRP, adeF, oqxA, baeR, K. pneumoniae KpnE

Table S4. Virulence and transposases genes detected in *K. pneumonia* and *E. coli* isolates

ID	Human pathogen probability	Virulence	Transposases
1EE	90	hra, gad, terC, astA, lpfA, terC	MITEEc1, MITEEc1, Tn6024, MITEEc1, ISPpu12, ISEcl1, MITEEc1, IS30, ISKpn8, IS4, IS421, IS629, ISKpn14.
4EE	88	terC, fyuA, irp2, lpfA, cia, iss, iss, ompT, gad, gad, terC, capU	ISSfl10, IS26, MITEEc1, MITEEc1, IS30, IS30, ISEc40, ISEc40, ISEc38, IS421, ISEc53, IS903, IS903
6EE	93	terC, lpfA, gad	MITEEc1, MITEEc1, IS421, MITEEc1, IS30, ISEsa1, IS609, MITEEc1
7EE	94	astA, terC, gad, iss,terC, lpfA	MITEEc1, MITEEc1, IS102, MITEEc1, MITEEc1, IS102, MITEEc1, ISEc31
11EE	94	terC	MITEEc1, ISKpn8, IS421, ISEsa1,
13EE	94	ompT, gad, lpfA, terC, iss	MITEEc1, ISEc17,
14EE	94	terC, etsC, papC,iss, iroN, lpfA, gad, iss, mchF	MITEEc1, IS679, ISKpn24, ISKpn24, IS682, IS30, cn_51933_IS679, ISEc46
16EE	94	terC, cia, gad, gad, lpfA	ISVsa3, ISKpn19, MITEEc1, IS102, ISEsa1, IS609, ISEam1, IS30, IS26
1EP	93	sitA, terC, senB, cea, lpfA, astA, capU, traT, iss, fyuA, irp2, ompT, iutA, iucC, cia, iss, terC	IS911, ISEc38, ISKpn24, ISEc30,
5EP	91	sitA, irp2, fyuA, kpsMII_K5, kpsE,aggC, ORF4, aatA, aggR, terC, gad, iss, eilA, air, chuA, capU, hra, traT	MITEEc1, ISVsa3, IS6100, ISEc46, ISEc9, ISEc38, IS30, MITEEc1, IS609, IS609, ISEc31, ISEc38, IS100, IS5075, IS5075, SEc48.
8EP	93	sitA, terC, eilA, kpsMII_K4, terC, hra, irp2, fyuA, sat, kpsE, iutA, iucC, senB, gad, traT, chuA, papA_F43	IS6100, ISEc9, Tn5403, IS421, ISKpn8, ISKpn8, ISEc38,
10EP	89	gad, kpsE, kpsMII_K1, irp2, fyuA, papC, papA_F12, terC, traT, iss	IS6100, ISEc43, ISEc38, ISEcB1, ISKox3, ISEc30, ISEc1, IS682, IS421, IS26, MITEEc1
11EP	93	sitA, iucC, traT, lpfA, air, fyuA, irp2, eilA, eilA, gad,chuA, terC, kpsE, yfcV, terC	IS6100, ISEc81, ISKpn8, ISEc38, ISEc30, IS421, ISEc45,
12EP	93	lpfA, terC, iss, ompT, terC	IS3, MITEEc1, ISSen4,
15EP	93	sitA, cnf1, fyuA, clbB, gad,focCsfaE, sfaD,chuA, iss, pic, vat, hra, terC, iss, iroN, kpsE, yfcV, cea, mchF, kpsMII_K23, usp, tcpC	ISEc42, MITEEc1, ISEc17, ICEEcIHE3034-1, ISEc38, ISEc13, ISEc41, IS100, ISEc38, ISEc31

20EP	93	cia, kpsE, kpsMII_K5, gad, eilA, traT, irp2, hra, chuA, papA_F19, terC, fyuA	Tn2, IS4, ISEc30, IS100,IS26
24EP	93	traT, kpsMII_K5, kpsE, eilA, terC, chuA, irp2, fyuA, cia, gad, hra, terC	IS6100, IS4, ISEc30, IS100, ISKpn8, IS26,
25EP	93	terC, eilA, hra, kpsE, kpsE, irp2, fyuA, chuA, papA_F19, gad, cia, traT	Tn2, IS4, IS100, ISEc30, IS26,
26EP	94	astA, afaD, eilA, air, aap, ORF3, iutA, iucC, capU, terC, hra, traT, chuA	MITEEc1, ISSpu2, IS629 cn_7104_IS629, IS911,IS682,ISEc30, MITEEc1, ISEc1, IS609, IS100, ISEc31, ISEc38, ISEc46
27EP	93	iha, iha, traT, terC, afaC, afaD, iss, fyuA, irp2, gad, terC, hra	IS6100, MITEEc1, ISKpn8, IS609, ISEc45, IS30, IS421, ISEc10, IS3, IS102,ISKox3
28EP	93	traT, gad, gad, irp2, fyuA, terC	IS6100, MITEEc1, ISKpn8, IS4, IS30, IS3, ISKpn26, IS26
30EP	93	eilA, air, chuA, fyuA, irp2, terC, kpsE, gad, terC, traT	IS102, , ISEc38, ISEc17, Tn5403, MITEEc1, ISEc1, IS609, IS4, cn_3324_ISEc1, ISEc30, ISKpn8, IS5075
1KE	90	iutA, terC, traT	ISEc9, IS6100, ISKpn24, ISEc30, ISKox1, ISEsa1, ISSm1, IS5075, ISKpn28, IS26, Tn3
2KE	91	iutA	ISSty2, ISSen4, ISKpn43, ISSpu2,
7KE	92	iutA, terC, traT	S903, SEc15, SKpn21, SKpn1, Tn3
8KE	92	iutA	SKox1, SEc52,
11ke	90	iutA, traT	SSty2, SEc52, SKpn14, SKpn24, SKpn38, SKpn28, SKpn1,
12ke	89	iutA, terC, traT	Tn6082, ISKpn43, Tn5403, ISEhe3, ISKpn38, ISEsa1, ISSty2
15ke	91	iutA, terC	ISEsa1, ISKpn14, ISKpn47, ISKpn43, IS102, ISKpn26, ISCfr26, ISKpn28
16ke	80	iutA	ISKpn41, ISKpn28,
17ke	90	iutA	ISSty2, ISKpn43, IS5075,
19ke	90	irp2, fyuA, iutA, traT	ICEEcoED1a-1, ISKpn26, IS26, ISEam1, ISKpn14, ISKpn1
20ke	90	iutA, traT	ISEhe3, ISKpn8, IS26, ISSen4, ISKpn34
21ke	90	iutA	NIL
23ke	89	iutA	ISEc33, ISEc52, ISSty2, ISKpn1,
3KP	92	iutA, traT	SEhe3, SSm1, S5075, SKpn42, SKpn14, SKpn21, SKpn38, SKpn43
5KP	92	iutA, traT	S5075,SEc9, SEhe3, SEcl1, SKpn1, SEc15, S6100, Tn3
6KP	90	iutA, traT	S5075, SEc9, SEc15, SKpn1, SEhe3, SEcl1, S6100, Tn3
7KP	89	iutA, traT	S5075, SEc9, SKpn1, SEhe3, SEcl1, SEc15

12kp	81	iutA, traT	IS5075, ISEc9, ISKpn1, IS6100, ISEc15, ISEhe3, ISEcl1
13kp	90	iutA, traT	IS5075, ISEc9, IS6100, ISEhe3, ISKpn1, ISEcl1, ISEc15, Tn3
14kp	90	iutA, traT	IS5075, ISEc9, ISEcl1,ISEhe3, ISEc15, IS6100, Tn3

Table S5. Co-occurrence of antimicrobial-resistance genes and transposases.

ID	Sample Source	Contig number	Contig length (Coverage)	ARG		IS/Tn		Plasmid	
				Name	position	Name	Position	Name	Position
1EP	Wound of postoperative infection	33	15007 (124)	*qnrB4*	1044-400	IS6100	10897-11776		
				mph(A)	14034-14939				
				qacE	7614-7824				
				sul1	7884-8723G				
				blaDHA-1	5168-6307				
8EP	UTI	652	9813 (30)	*dfrA17*	1224-751	IS6100	5703-6582		
				qacE	2349-2630				
				mph(A)	8840-9745				
				sul1	2690-3529				
				aadA5	1355-2143				
		381	5965 (31)	*bla*$_{CTX-M-15}$	2453-3328	**ISEc9**	749-2404		
		131	17556 (264)			**Tn5403**	47-3694	IncFII(29)	6928-7186
10EP	Wound of postoperative infection	79	**9975 (457)**	***mph(A)***	9004-9906	IS6100	5855-6734		
				dfrA17	1376-903				

				sul1	2842-3681				
				aadA5	1507-2295				
11EP	Wound of	257	**9949 (100)**	*qacE*	7231-7512	IS6100	3279-4158		
	postoperative infection			*dfrA17*	8637-9110				
				mph(A)	1021-116				
				sul1	6332-7171				
				aadA5	7718-8506				
14EE	Water	13	**126908 (264)**			**IS679**	72830-75533	IncFII	121336-121595
						**ISKpn24**	79321-81774		
						**IS682**	24433-26966		
						**IS30**	10801-12021		
						**cn_51933 IS679**	23600-75533		
16EE	Water	45	5269 (231)	*qnrS1*	3599-4255	**ISKpn19**	1-2850		
		48	3983 (254)	*floR*	1541-328	**ISVsa3**	2660-3636		
		42	13543 (230)			**IS102**	37-1092	p0111	2936-3820
20EP	Urine (Chronic Kidney Disease)	224	8722 (222)	*rmtB*	1461-706	Tn2	1484-6433		
				bla$_{TEM-1B}$	1631-2491				
24EP	sepsis	1370	4166 (41)	*mph(A)*	3195-4097	**IS6100**	46-925		

25EP	Urine (Chronic Kidney Disease)	401	8726 (142)	*rmtB*	1461-706	Tn2	1484-6433		
				bla$_{TEM-1B}$	1631-2491				
27EP	UTI	580	11125 (169)	*mph(A)*	1022-117	IS6100	3280-4159		
				qacE	7232-7513				
				dfrA17	8638-9111				
				sul1	6333-7172				
				aadA5	7719-8507				
28EP	Recurrent UTI	165	21847 (142)	*mph(A)*	1023-118	IS6100	3281-4160		
				bla$_{TEM-35}$	5141-6001				
30EP	UTI	77	6551 (306.4)	tet(B)	1898-3103	**IS102**	3114-4170		
		66	11302 (252.7)			**Tn5403**	506-4169	IncFII	7404-7664
1KE	Vegetables	33	12084 (250.6)	*bla*$_{TEM-1D}$	6334-7194	IS1380-like, ISKra4-like, **Tn3**	3562-5217 7400-8400 1-2350	PRiA4b ORF3	9050-9650
				qnrS1	10976-11632				
				bla$_{CTX-M-15}$	2638-3513				
		34	5958 (241.1)	*mph(A)*	4984-5889	**IS6100**	1847-2726		
2KE	Surface	16	130723 (310.9)	*OqxB*	10870-7718	ISSty2	2245-3503		
				OqxA	6519-7694				
5KP	Neonatal sepsis	361	2832 (183)	*sul2*	1982-2797	IS5075 IS91	223-1549		
				aph(6)-Id					
				aph(3'')-Ib					
		334	5415 (178)	*bla*$_{CTX-M-15}$	2506-3381	**ISEc9, Tn3**	802-2457 3786 -6005		

6KP	Neonatal sepsis	522	5098 (163)	*aph(6)-Id*	3661-4497	IS5075<br>IS91	223-1549		
				aph(3'')-Ib	2858-3661				
				sul2	1982-2797				
		281	6016 (171)	*bla*$_{CTX-M-15}$	2684-3559	ISEc9	3608-5263		
						Tn3	2279 - 60		
7KP	Sepsis	140	5098 (197)	*aph(6)-Id*	1486-650	IS5075<br>IS91	3598-4924		
				aph(3'')-Ib	1486-2289				
				sul2	2350-3165				
		68	1641 (301)	*bla*$_{CTX-M-15}$	2684-3559	ISEc9	3608-5263		
						Tn3	1-2275		
11KE	Vegetables	1	501618 (228)	*bla*$_{SHV-1}$	27779-28645	**ISSty2**	459960-461218		
12KE	Fruits	303	13004 (100)	*aph(3'')-Ib*	8477-9279	Tn3,	1100-3900, 10100-12900		
				aph(6)-Id	7641-8477	IS110	1-1000		
13KP	Neonatal sepsis	210	5098 (193)	*aph(6)-Id*	1486-650	IS5075<br>IS91	3598-4924		
				aph(3'')-Ib	1486-2289				
				sul2	2350-3165				
		156	6016 (249)	*bla*$_{CTX-M-15}$	2684-3559	**ISEc9**<br>**Tn3**	3608-5263,<br>5077 -3815		
14KP	Neonatal sepsis	211	5098 (267)	*aph(6)-Id*	3661-4497	IS5075,<br>IS91	223-1549		
				aph(3'')-Ib	2858-3661				

				sul2	1982-2797				
		256	3312 (277)	*bla*$_{CTX-M-15}$	1-855	**ISEc9Tn3**	904-2559		
15KE	Surface in Market	35	10536 (306)			**ISKpn14**	3140-3907	IncHI1B	8736-9305
16KE	Surface in Market	11	133532 (143)			**ISKpn41**	4042-5467	IncFIB(Mar)	36801-37239
20KE	vegetables	34	38475 (784)			**ISEhe3**	35866-37094	repA	31937-32627

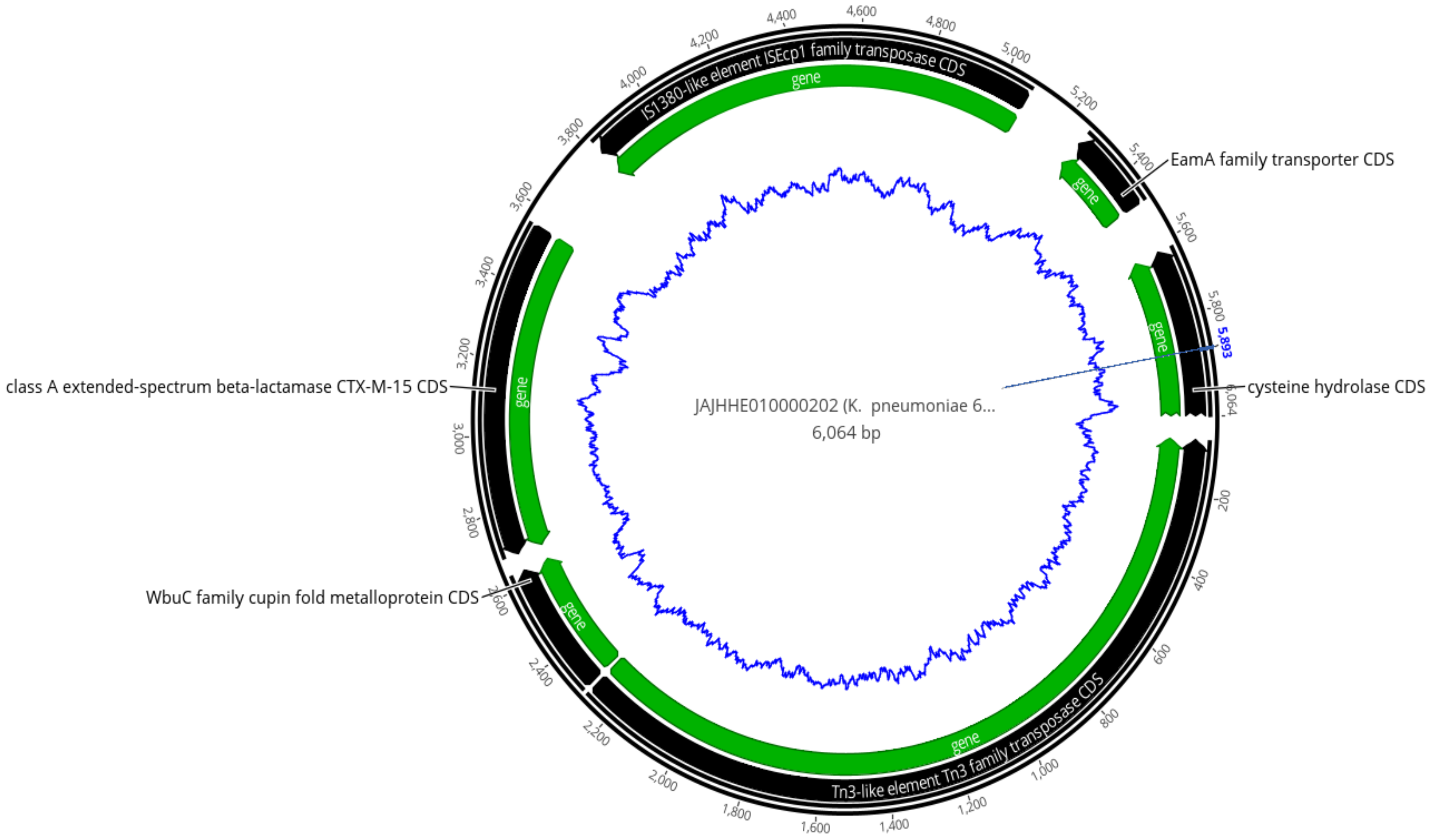

Figure S1. Map of different antibiotic-resistant genes, transposases, and plasmid, clustered in contig 202 of clinical *K. pneumonia* (5KP) isolate. Showing an example for the presence of *CTX-M-15* gene between two transposases. The outer black circle indicates for the length of the contig, black arrows indicate for coding sequences (CDS), green arrows indicate genes, and blue inner zigzag circle indicates GC content.

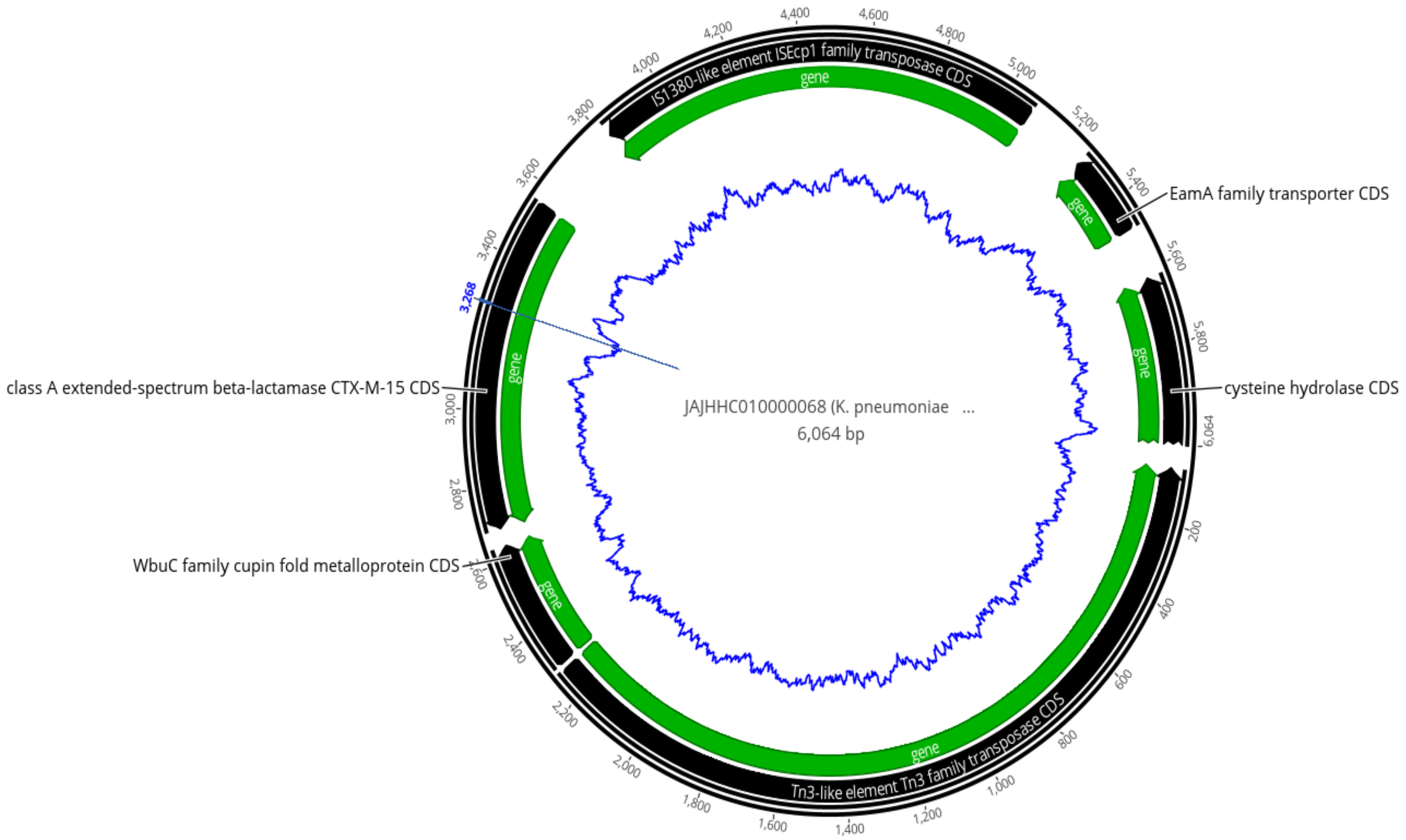

Figure S2. Map of different antibiotic resistant genes, transposases, and plasmid, clustered in contig 68 of clinical *K. pneumonia* (7KP) isolate. Showing an example for the presence of *CTX-M-15* gene between two transposases. The outer black circle indicates for the length of the contig, black arrows indicate for coding sequences (CDS), green arrows indicate genes, and blue inner zigzag circle indicates GC content

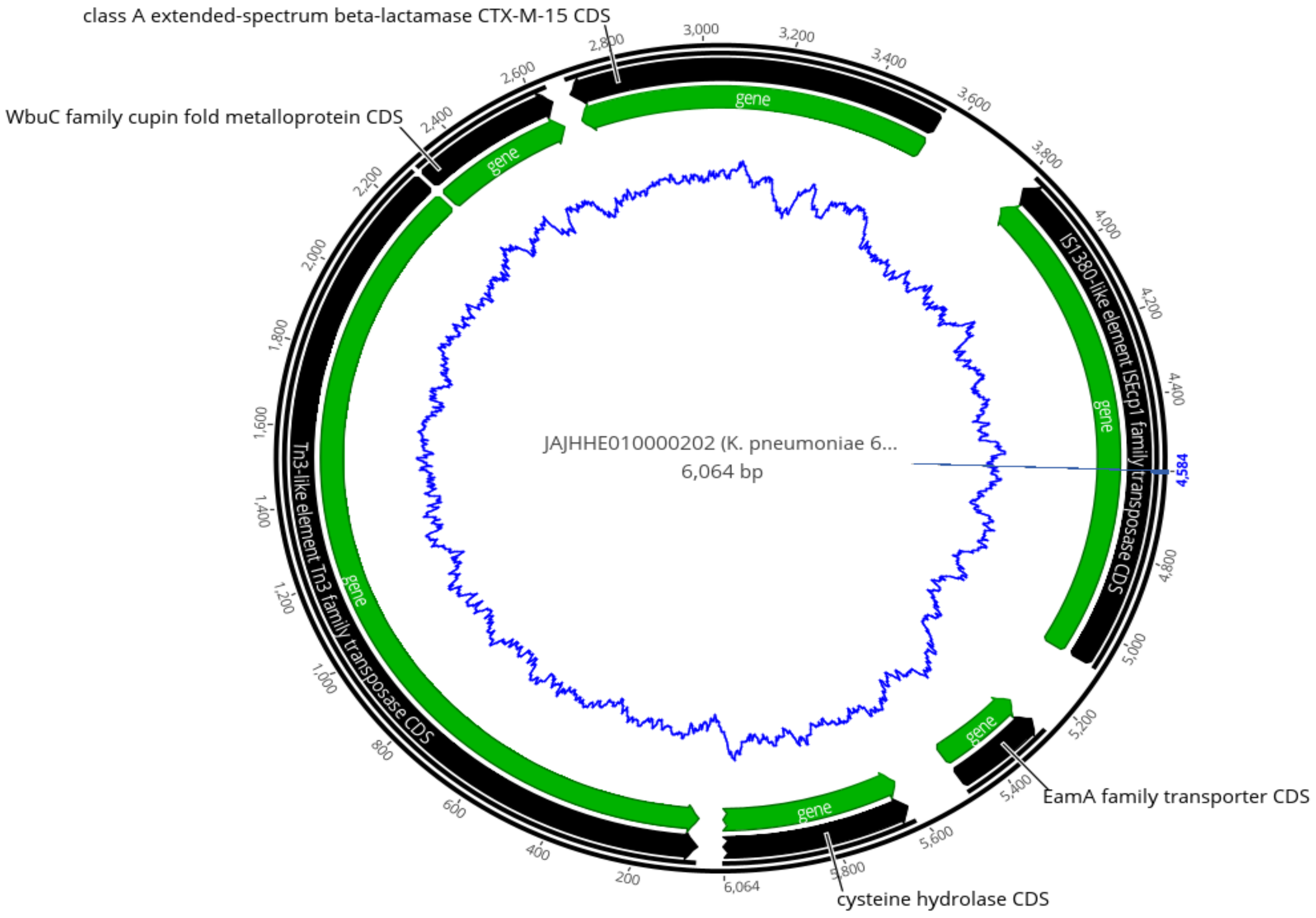
class A extended-spectrum beta-lactamase CTX-M-15 CDS
WbuC family cupin fold metalloprotein CDS
IS1380-like element ISEcp1 family transposase CDS
Tn3-like element Tn3 family transposase CDS
EamA family transporter CDS
cysteine hydrolase CDS
gene
JAJHHE010000202 (K. pneumoniae 6...
6,064 bp
4,584

Figure S3. Map of different antibiotic resistant genes, transposases, and plasmid, clustered in contig 202 of clinical *K. pneumonia* (6KP) isolate. Showing an example for the presence of *CTX-M-15* gene between two transposases. The outer black circle indicates for the length of the contig, black arrows indicate for coding sequences (CDS), green arrows indicate genes, and blue inner zigzag circle indicates GC content

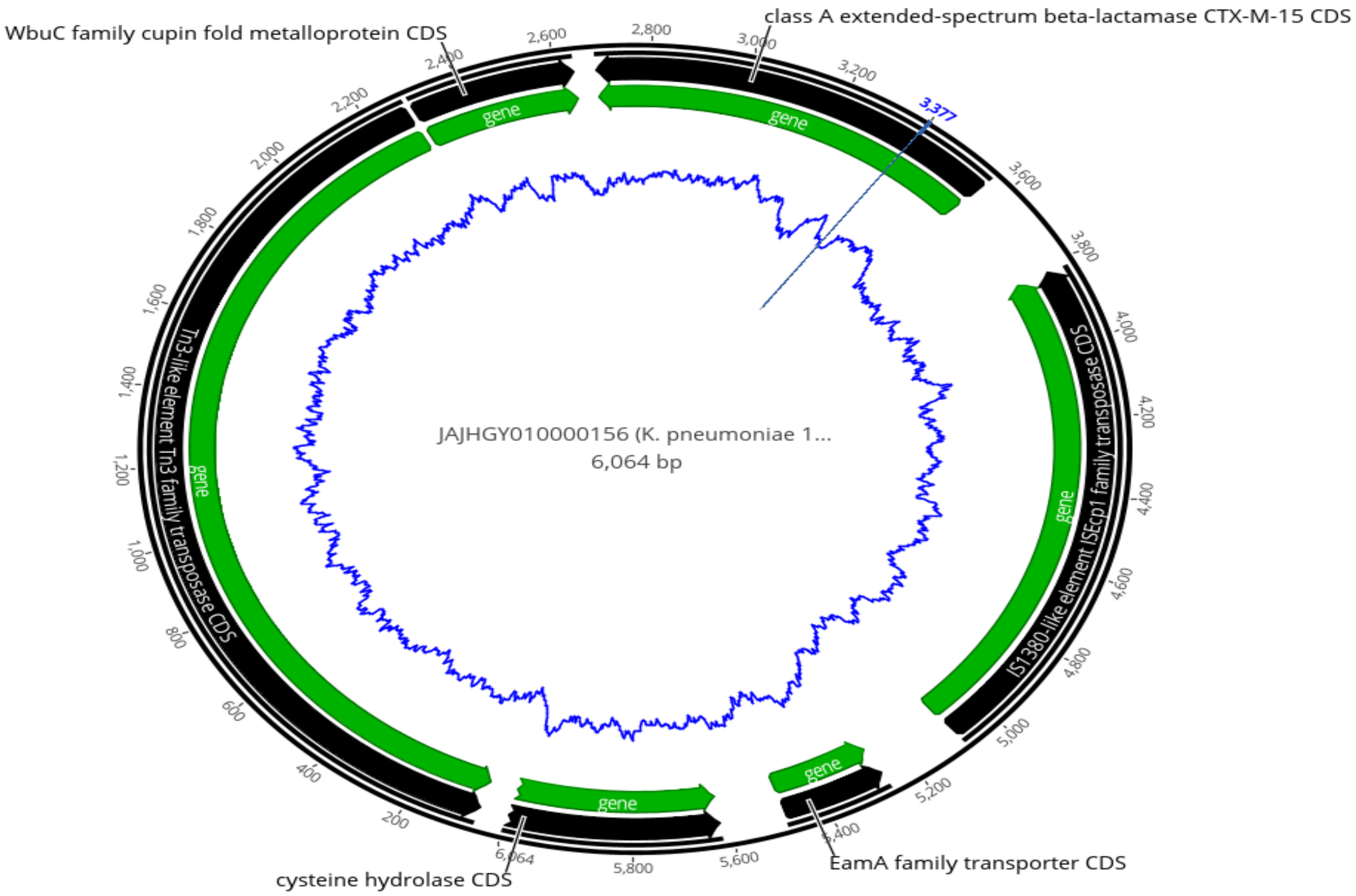

Figure S4. Map of different antibiotic resistant genes, transposases, and plasmid, clustered in contig 156 of clinical *K. pneumonia* (13KP) isolate. Showing an example for the presence of *CTX-M-15* gene between two transposases. The outer black circle indicates for the length of the contig, black arrows indicate for coding sequences (CDS), green arrows indicate genes, and blue inner zigzag circle indicates GC content.

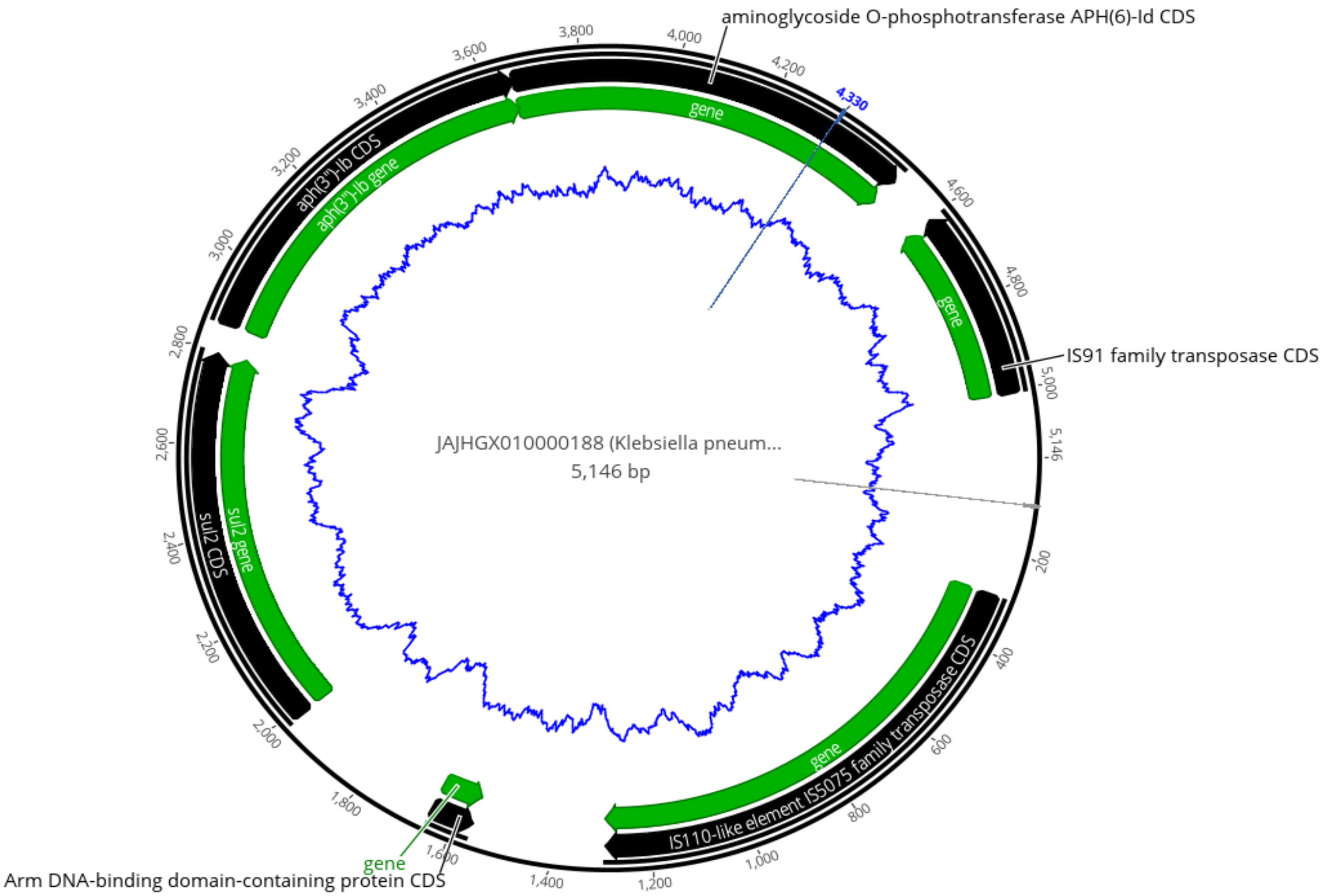

Figure S5. Map of different antibiotic resistant genes, transposases, and plasmid, clustered in contig 156 of clinical *K. pneumonia* (14KP) isolate. Showing an example for the presence of aminoglycosides genes flanked by transposases. The outer black circle indicates for the length of the contig, black arrows indicate for coding sequences (CDS), green arrows indicate genes, and blue inner zigzag circle indicates GC content
